# The rectal vaginal opacification with water and the antiperistaltic agent in magnetic resonance scanning of the intestinal endometriosis

**DOI:** 10.4274/tjod.galenos.2019.43788

**Published:** 2019-10-10

**Authors:** Cemil Gürses, Baris Mulayim, Mete Çağlar

**Affiliations:** 1 University of Health Sciences, Antalya Training and Research Hospital, Clinic of Radiology, Antalya, Turkey; 2 University of Health Sciences, Antalya Training and Research Hospital, Clinic of Obstetrics and Gynecology, Antalya, Turkey; 3 Akdeniz University Faculty of Medicine, Department of Obstetrics and Gynecology, Antalya, Turkey

**Keywords:** Endometriosis, ultrasound, magnetic resonance imaging, menstruation, painful

## Abstract

The diagnosis of deep intestinal endometriosis is mandatory to plan treatment and for follow-up; however, there is no consensus worldwide in the use of rectal/ vaginal opacification and anti-peristaltic agents for magnetic resonance imaging (MRI) scanning, being defined as an option for the examination. The transvaginal ultrasound images of previous MRI with the standard protocol, and recent MRI in our institution with rectal/vaginal opacification with water and the anti-peristaltic agent are presented in four cases for comparison, respectively. The technique in our institution seems to be more effective than routine pelvic MRI scans in the intestinal endometriosis.

## Introduction

Deep infiltrating endometriosis (DIE) is involvement of the retrocervical septum, rectovaginal septum, uterosacral ligaments, vaginal fornix, and bladder^(1)^, and intestinal involvement occurs in 12-37% of patients with endometriosis^(2)^. Transvaginal ultrasound (TVUS) is the first-line method and magnetic resonance imaging (MRI) should be considered as a second-line technique after TVUS^(3)^. However, the MRI diagnosis of DIE is a dilemma in radiology departments because most MRI scans with suspicion of DIE are negative in spite of either an intestinal endometriotic nodule in TVUS or the clinical history being in favor of DIE. There is no universally accepted protocol for MRI in endometriosis for the use of vaginal and/or rectal opacification (RVO)^(3).^ In the presented cases, which were initially diagnosed using TVUS as intestinal endometriosis, the findings of MRI scans with and without RVO with water and anti-peristaltic agent use are presented.

## Case Report

The 53 patients with TVUS findings in favor of deep pelvic endometriosis were collected between 2016 and 2018. The criterion for patient selection was the presence of at least three of the following TVUS findings: ^(1)^ restriction of uterus mobility or pain with probe compression; ^(2)^ kissing ovaries; ^(3)^ unilateral or bilateral ovarian endometrioma;^ (4) ^intestinal wall thickening; and ^(5)^ intestinal endometriotic nodule either as the mushroom cap sign or the Indian headdress sign^(4)^.

Twenty-nine patients who were examined using MRI due to TVUS findings and the previous MRI scans of 28 patients, performed either in our center or an external tertiary care hospital or in a private hospital, were re-examined retrospectively. All of the MRI scans were reported to be normal concerning DIE in spite of clinical information or TVUS findings. Four of the 28 patients with endometriosis with intestinal involvement considering the TVUS findings were re-scanned using MRI prospectively due to serious clinical symptoms. All of the MRI re-scans were performed according to the European Society of Urogenital Radiology DIE guideline^(3)^. Written informed consent was obtained from all subjects, according to the World Medical Association Declaration of Helsinki, revised in 2000, Edinburgh. The histories and the clinical information of the 4 patients are obtained as suggested by the IDEA group^(4)^ and these were noted for each patient respectively (Table 1).

### Technique

**Figure 1:**
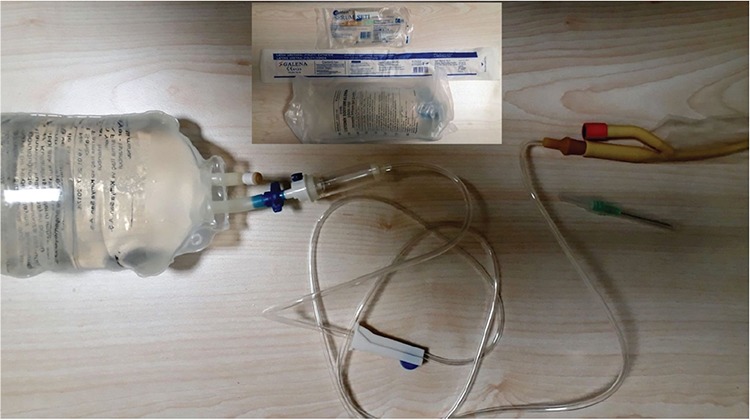
The simple system for administering the water into the urinary bladder (if needed), the vagina and the bowel lumen. The same system is used for the voiding cystourethrography

A 1.5 Tesla Magnetom_Essenza MRI system (Siemens AG Wittelsbacherplatz 2 80333 Muenchen Germany) was used for the MRI re-scans anda Toshiba Applio 500 ultrasound system was used for transvaginal examinations (TUS-A500, Toshiba Medical Systems, Europe BV, Zilverstraat 1, 2718 RP, Zoetermeer, The Netherlands). The patients were informed to use a rectal enema 12 and 2 hours before the MRI exams for distal bowel cleansing. If the urinary bladder filling was inadequate in the survey image, 200 mL of saline infusion was administered through a Foley catheter. Isotonic saline solution was used for vaginal and RVO through the Foley or Nelatone urinary catheter. The vaginal filling with saline infusion was stopped when the patient started to feel overflow. The total amount of fluid used for rectal filling was between 500 mL and 1000 mL. After the intravenous administration of the single-dose (20 mg) American Psychological Association (APA) (Hiyosin N Butilbromur, Buscopan, Zentiva), 100-200 mL of additional fluid for bowel filling was given. The urinary bladder (if needed), vaginal and rectal saline infusions were easily performed with a simple and cheap system (Figure 1).

### Magnetic Resonance Imaging Sequences

**Figure 2:**
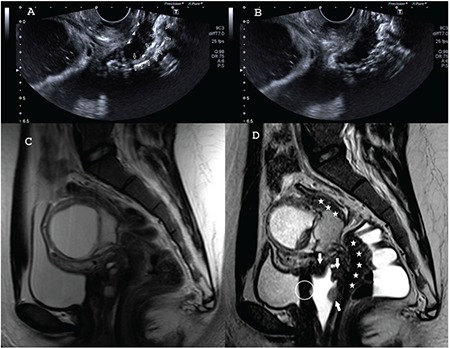
A) Transvaginal ultrasound (TVUS) in patient 1. The mushroom cap sign: the arrows represent the cap, which consists of the mucosa and submucosa, the arrowheads represent the base of the mushroom, which consists of fibrosis of the muscular propria. The mushroom cap sign has been described on TVUS and magnetic resonance imaging (MRI) and is accepted as a characteristic finding of severe involvement in deep infiltrating endometriosis, B) TVUS in patient 1. The Indian headdress or moose antler sign; the arrows represent the spiky extensions of the fibrosis of the muscular propria towards the bowel lumen, C) Sagittal T2W image of patient 1 in the previous MRI scan. Vaginal active bleeding and the sigmoidal chronic fibrotic lesions might be noticed only by experienced eyes, D) Sagittal T2W image of patient 1 in the present MRI scan. The vaginal and the sigmoidal lesions can be detected easily by all examiners. The white arrows represent the vaginal and the white stars represent the sigmoidal lesions. The circle indicates the invasion of the vaginal wall fibrosis into the urinary bladder. This will be shown in the following T1W Dixon image with the white arrows

**Figure 3:**
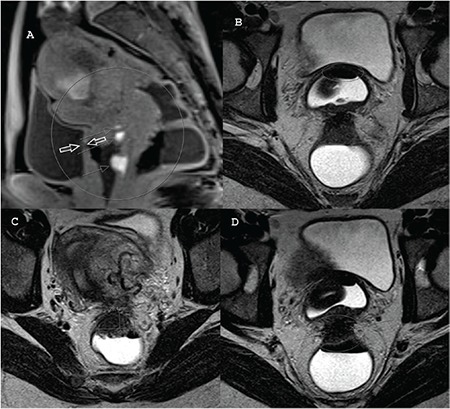
A) T1W Dixon image of patient 1 in the present magnetic resonance imaging (MRI) scan. The white arrows represent the invasion of the posterior wall of the urinary bladder by the vaginal anterior wall fibrosis. The thin arrows indicate the posterior vaginal wall lesions with subacute bleeding, B) Axial T2W image of patient 1 in the present MRI scan. The circle shows the fibrotic invasion between the urinary bladder and the vagina, C) Axial T2W image of patient 1 in the present MRI scan shows the measurement of transverse diameter of the anterior sigmoidal wall lesion, D) Axial T2W image of patient 1 in the present MRI scan. The circle and the thin arrows show the fibrotic invasion between the sigmoid bowel and the vagina

**Figure 4:**
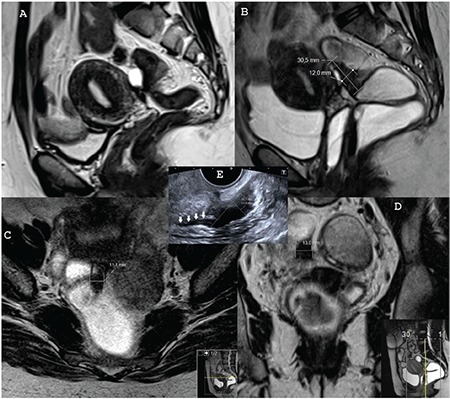
A) Sagittal T2W image of patient 2 in the previous magnetic resonance imaging MRI scan. It is not possible to detect the actual place of the intestinal involvement, B) Sagittal T2W image of patient 2 in the present MRI scan. The intestinal involvement of the endometriosis in the sigmoid bowel, which was measured, can be detected easily, C) Axial T2W image of patient 2 in the present MRI scan. The lesion is measured in a horizontal plane, D) Coronal T2W image of patient 2 in the present MRI scan. The lesion is measured, E) Transvaginal ultrasound in patient 2. The endometriotic intestinal nodule is measured. The nodules due to the hypertrophy and the fibrosis of the muscular propria grow into the lumen and narrowing the diameter. The nodule has a comet shape with a tail; comet sign

**Figure 5:**
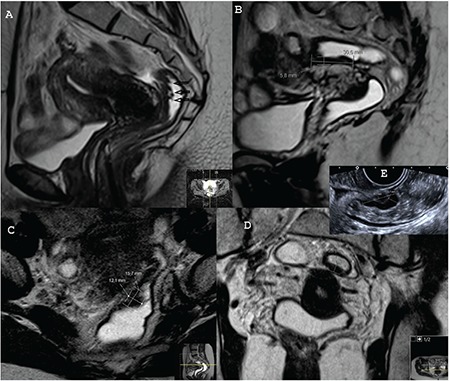
A) Sagittal T2W image of patient 3 in the previous magnetic resonance imaging (MRI) scan. There seems to be wall thickening with an Indian headdress sign (arrows). However, after the administration of the rectal fluid and the anti-peristaltic agent, the actual lesion is found in another segment of the sigmoid bowel, B) Sagittal T2W image of patient 3 in the present MRI scan. The actual place of the sigmoid bowel involvement was in a different segment in contrast to the previous MRI scan (Figure 5A), C) Axial T2W image of patient 3 in the present MRI scan. The lesion is measured, D) Coronal T2W image of patient 3 in the present MRI scan. The lesion is measured, E) Transvaginal ultrasound in patient 3. The endometriotic intestinal nodule is measured. There is regular thickening in the muscular propria

**Figure 6:**
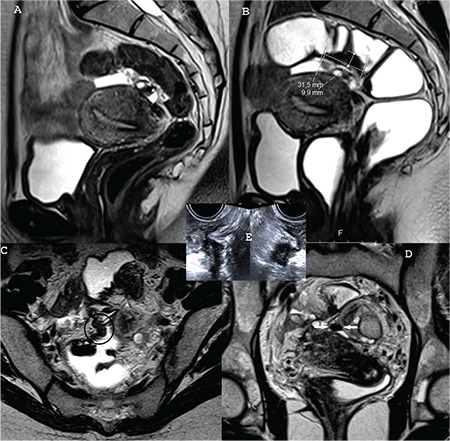
A) Sagittal T2W image of patient 4 in the present magnetic resonance imaging (MRI) scan obtained initially just before applying the rectal/vaginal opacification with water and the anti-peristaltic agent in our tertiary care hospital, but with the rectal cleansing enema, B) Sagittal T2W image of patient 4 in the present MRI obtained with the rectal/vaginal opacification with water and the anti-peristaltic agent use after the lesion is measured, C) Axial T2W image of patient 4 in the present MRI obtained with the rectal/ vaginal opacification with water and the anti-peristaltic agent use. The lesion is in the circle, D) Coronal T2W image of patient 4 in the present MRI obtained with the rectal/vaginal opacification with water and the anti-peristaltic agent use. The lesion is between the arrows, E) Transvaginal ultrasound in patient 4. The endometriotic intestinal nodule is measured. There is irregular thickening in the muscular propria

2D T2W sagittal/axial/coronal, 2D T1W sagittal/axial with and without fat saturation, T1W Dixon sagittal, and diffusion-weighted sequences were performed. Gadolinium is not used for intravenous or in saline solution for contrast opacification. If there was an interval more than 3 months between the previous and the present MRI scan, initial T2W sagittal images were obtained just before applying the RVO with water and the APA in order to exclude recent nodule growth. The findings in TVUS, the previous MRI scans, and the re-scanned MRI examinations are presented as images, respectively. The endometriotic nodules in the bowel wall are demonstrated clearly in all patient re-scans, which had been reported as normal previously (Figure 2, 3, 4, 5, 6).

## Discussion

The MRI examinations are crucial for the proper diagnosis and for the demonstration of the extent of the lesions preoperatively, for postoperative follow-up or for the efficacy of medical treatment. The MRI findings of endometriotic nodules in the intestinal wall vary depending on the progression of the infiltration. On T1W sequences, the lesions are hyperintense in early phases due to bleeding and hypointense in the chronic phases due to fibrosis^(6)^. Mostly the lesions are in the chronic phase, which is why the bright intestinal lumen is mandatory in order to visualize the fibrotic nodules, especially for inexperienced examiners. However, there is no consensus for RVO with water and APA use, which is published to be an option in the medical literature^(3,7)^. In daily practice, MRI scans are performed without these options. Here, it is clearly seen that MRI with RVO with water and APA use is more appropriate than routine MRI scans for intestinal DIE. Administration of the optional procedures requires an extra 10 to 15 minutes, so it might be not preferred in some radiology departments. In Turkey, public hospitals are autonomized to allow them to out-source some medical services such as diagnostic imaging,^(8)^ and the less time required for MRI scanning means more income for the out-source services. Therefore, the use of the optional technique should be restricted by choosing patients with DIE before the MRI scans using TVUS. In our experience, the diagnosis of intestinal involvement in endometriosis using MRI needs RVO with water and APA use; therefore, it should not be an option in MRI scans as in the guidelines, but an obligation in patients with endometriosis with intestinal involvement in order to increase the detectability.

## Figures and Tables

**Table 1:**
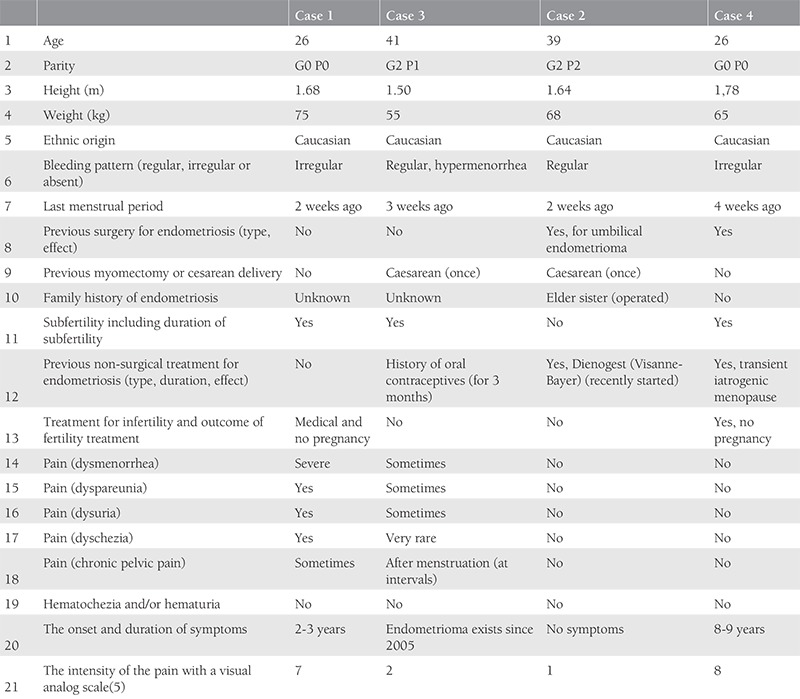
The clinical information obtained for each patient
